# Explaining the sex difference in depression with a unified bargaining model of anger and depression

**DOI:** 10.1093/emph/eow006

**Published:** 2016-02-15

**Authors:** Edward H. Hagen, Tom Rosenström

**Affiliations:** ^1^Department of Anthropology, Washington State University, 14204 NE Salmon Creek Avenue, Vancouver, WA 98686, USA; ^2^Institute of Behavioural Sciences, University of Helsinki, Helsinki 00014 (PO Box 9), Finland

**Keywords:** depression, grip strength, bargaining, sex difference

## Abstract

**Background**: Women are twice as likely as men to be depressed, a bias that is poorly understood. One evolutionary model proposes that depression is a bargaining strategy to compel reluctant social partners to provide more help in the wake of adversity. An evolutionary model of anger proposes that high upper body strength predisposes individuals to angrily threaten social partners who offer too few benefits or impose too many costs. Here, we propose that when social partners provide too few benefits or impose too many costs, the physically strong become overtly angry and the physically weak become depressed. The sexual dimorphism in upper body strength means that men will be more likely to bargain with anger and physical threats and women with depression.

**Methodology**: We tested this idea using the 2011–12 National Health and Nutrition Examination Survey (NHANES), a large nationally representative sample of US households that included measures of depression and upper body strength.

**Results**: A 2 SD increase in grip strength decreased the odds of depression by more than half (OR=0.4, P=0.0079), which did not appear to be a consequence of confounds with anthropometric, hormonal or socioeconomic variables, but was partially explained by a confound with physical disability. Nevertheless, upper body strength mediated 63% of the effect of sex on depression, but the mediation effect was unexpectedly moderated by age.

**Conclusions**: Low upper body strength is a risk factor for depression, especially in older adults, and the sex difference in body strength appears to explain much of the perplexing sex difference in depression.

## Abstract (precis)

Most studies of depression find that women are twice as likely to be depressed as men, but no compelling explanation of this sex difference has emerged. Here, we show that the sex difference in depression can largely be explained by the sexual dimorphism in upper body strength.

## Conflict over division of benefits

Human groups are characterized by a high level of cooperation. Theoretical and empirical evidence indicates that such cooperation faces constant challenges, such as free-riding and ‘unfair’ allocation of the fruits of cooperation. These challenges represent selection pressures for individual counter-strategies to identify, punish, and otherwise thwart excessive diversion of benefits to other group members [1].

Adversity, which we define as circumstances that have the potential to reduce an individual's biological fitness, is an under-appreciated source of conflict, which we define as a situation where one agent’s strategy to increase her fitness causes a decrease in the fitness of another agent. Adversity facing the entire group, such as an external threat, usually reduces intra-group conflict and increases cooperation [2]. Adversity facing a single group member, however, can potentially increase conflict with all other group members because the victim often engages in strategies to increase her fitness that have the effect of decreasing the fitness of social partners. Examples of adversity causing conflict include illness, marital conflict, physical or sexual assault, or death of a spouse, all of which might increase help-seeking by the victim, requiring others to provide care, protection, alloparenting, and other forms of investment at an increased cost to themselves. Adversity can thus reduce the profitability of cooperative ventures for some, necessitating concessions by others if cooperation is to be maintained [3].

Hagen [3] suggested that physical aggression and depression were complimentary strategies to resolve conflicts over divisions of benefits among cooperative partners: physical aggression would be effective for those who were physically strong or had allies; depression, as we explain shortly, would be effective for those who were not physically strong and/or lacked allies.

## The bargaining model of anger

There is considerable evidence that ‘unfair’ divisions provoke anger and other negative emotions. The ultimatum game (UG) is a simple bargaining game in which the first mover offers a division of a fixed amount of money, k, to the second mover. If the second mover accepts the division both players receive their share; if she rejects it, neither player receives anything. Studies have found that in response to low ‘unfair’ offers in the UG, anger is elevated, second movers punish first movers with rejections and express negative emotions, and brain regions are activated that involve emotional responses, especially negative ones [4–7].

Sell *et al.* have found that, in humans, a propensity to become angry, and to have a history of physically fighting, is closely tied to upper body strength, especially in men [8–11]. In a study of US college students, male upper body strength was significantly positively correlated with proneness to anger, a history of fighting, belief in the utility of personal aggression, belief in the utility of political aggression, a sense of entitlement, and self-reported success in conflicts. Among women, in contrast, physical attractiveness, but not upper body strength, correlated with all of the foregoing (except a history of physical fighting) [9]. Among Aka hunter-gatherers, upper body strength predicted peer-rated anger, which in turn predicted peer-rated physical violence by both sexes [11]; similar relationships have been found in other non-Western populations [10, 12]. On this view, anger is part of bargaining strategy, backed by physical strength or physical attractiveness, to increase an individual's share of important resources and help resolve conflicts in his or her favor [8, 9].

## The bargaining model of major depression

Sadness or low or depressed mood is a common response to adversity, such as death of a loved one or divorce. Sadness and low mood are often accompanied by crying and facial expressions that are universally recognized as sad [13], which strongly suggests they are, in part, evolved signals of distress to social partners. This, in turn, suggests that individuals suffering adversity often need help from their social partners.

In response to adversity, a minority of individuals suffers major depression (MD), an affliction that involves prolonged negative affect and/or loss of interest in virtually all activities (most major depressive episodes—80–90%—occur in the wake of adversity [14]). Hagen [3, 15, 16] and Rosenström [17] argued that the prolonged loss of interest that characterizes most MD is an evolved bargaining strategy (a similar argument was made by [18]). When there is conflict in interdependent relationships, individuals experiencing adversity might not receive sufficient help from social partners. Loss of interest can serve as an unconscious means to withhold the benefits one provides to others until sufficient help is forthcoming. A mother of a newborn who is not receiving enough support from her husband or family, for example, might suffer postpartum depression and reduce investment in the baby, compelling the husband and family to invest more [15, 16]; she, in turn can then invest more in the new offspring. Similarly, other individuals who provide important benefits, such as grandparents, can withhold these benefits to compel desired changes in the behavior of, e.g. their adult children. Regarding a group context, Rosenström [17] identified depression with an additional option to ‘abstain’ or ‘not participate’ in ‘unfair’ interactions, which is a strategy that allows the evolution of cooperation in public goods games.

The bargaining hypothesis of MD is supported by the following well-established facts. First, sufferers of MD are often embroiled in interpersonal conflict [19, 20]. Second, they are frequently angry [21–23]. Third, they feel entrapped and powerlessness [24–26], and thus plausibly cannot respond to adversity without help. Fourth, depressed individuals have substantially reduced productivity [27], including in small-scale societies [28–30], and they cooperate less [31]. Fifth, social partners, despite conflicts and their negative reactions to MD [32], reduce aggression and increase positive reactions in response to depressive behaviors, so much so that many researchers worry these benefits reinforce depression [33–35]. Finally, in small kin-based societies, much (but not all) suicidal behavior, an important symptom of MD, is a response to conflict by powerless individuals that often elicits benefits if the victim survives [36, 37]. There are many other evolutionary theories of depression, most of which posit an evolved strategic response to adversity, and thus overlap with the bargaining model to some extent. For a critical review and comparison, see Ref. 38.

## The sex difference in major depression

MD is about twice as prevalent in women as men, with some cross-national variation [39–41]. Because this sex difference emerges in adolescence, some think hormonal changes during puberty, interacting with other biological and social factors, play an important role. Perhaps these changes, especially the onset of hormonal cycling in girls, result in sex differences in stress reactivity [42–44]. There is also evidence that testosterone might be protective against depression in adolescents and adults [45]. More generally, the affective and cognitive vulnerabilities thought to predispose to depression might differ by sex, and so too might exposure to negative life events, such as sexual abuse, that appear to be particularly potent causes of depression [46]. However, it is not clear that sex differences in exposure to negative life events explains the sex difference in depression [43]. Cross-national variation in gender equality is a possible cause of variation in the size of the sex difference in depression, although results have been inconsistent [40, 47]. In one study that parallels the hypothesis we discuss next, sex differences in anger appeared to explain the sex difference in depression [23].

## Hypothesis: the sex difference in physical formidability explains the sex difference in MD

Physical formidability and aggression are closely tied to upper body strength [8]. According to the bargaining model of anger, an angry threat is only credible for those with sufficient upper body strength or physical attractiveness relative to social partners [8, 9]. According to the bargaining model of depression, withdrawal of investment in joint ventures is only effective for those who provide valuable benefits to their social partners. Social value will often be independent of physical formidability. A young woman might have relatively low upper body strength, for example, but still be highly valuable to her parents and spouse. Hence, as suggested by Hagen [3], depression is an alternative strategy to physical threats for changing the behavior of recalcitrant social partners by withholding benefits.

**Figure 1.**
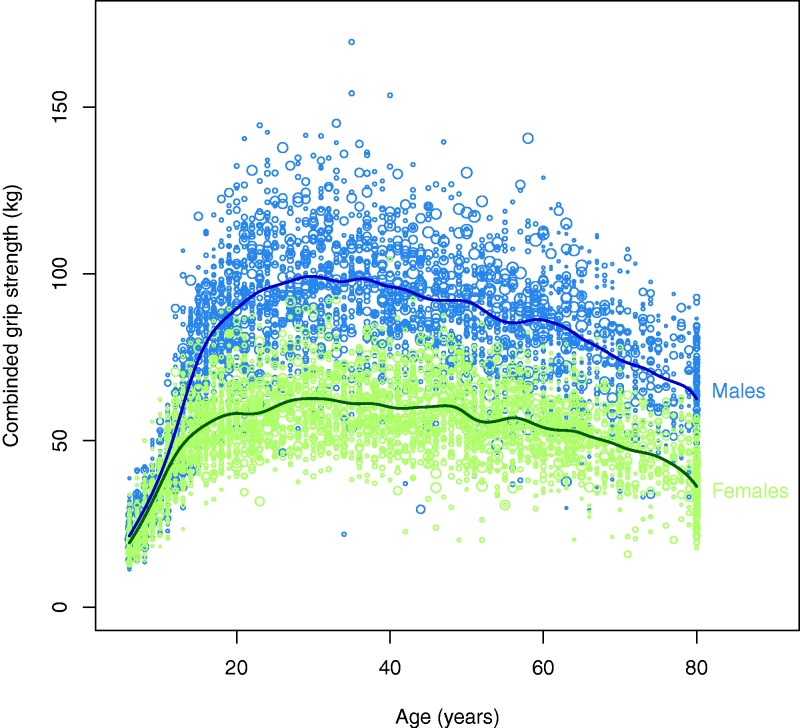
The relationship between combined grip strength and age. Each dot is one participant; bubble size is proportional to the survey weight of that datum. Males: blue (top). Females: green (bottom). Lines fit by local polynomial kernel smoothing using the survey package. Due to concerns over confidentiality, NHANES reports the age of all individuals over 80 as 80

Here, we test two interrelated hypotheses that follow from a unification of the two bargaining models. First, upper body strength, as an index of physical formidability, should be inversely related to depression. Second, the sex bias in depression is therefore due to the sexual dimorphism in body strength, especially upper body strength (Fig. 1) which is highly sexually dimorphic: across 112 data sets on adult sex differences in strength, the average ratio of female-to-male strength was 58% for upper limbs, 62% for trunk, and 66% for lower limbs. For upper limbs and trunk, 92% and 88% of men, respectively, would be stronger than women in chance encounters [48]. Importantly, the sex difference in upper body strength emerges in adolescence (Fig. 1), corresponding to the emergence of the sex difference in depression. Under the unified bargaining model, sexually dimorphic strength implies that men should be more likely to use overt physical threats and aggression to resolve conflicts (for evidence of a pervasive male bias in physical aggression, see [49]) and women should be more likely to use depression.

## Previous studies examining depression vs strength

According to Sell *et al.*, upper body strength is the anthropometric variable that best accounts for variation in overt anger and fighting in physically healthy adolescents and adults, and not height, weight, BMI, lower body strength or other indices of physical fitness or physical activity *per se* [9, 11, 50] (but see [12]). Although numerous studies have found a negative relationship between depression and physical activity in adolescents and adults [51], these do not illuminate the relationship between upper body strength and depression, nor do studies on muscle performance vs. depression in geriatric populations or in patients with physical illness.

Hand grip strength is an index of upper body strength. Several large studies have found a negative cross-sectional relationship between grip strength and depression levels in women and/or men, and some have also found that low grip strength predicted either persistence of depression symptoms or future depression symptoms [52–55]. None of these studies tested whether sex differences in upper body strength explained the sex difference in depression.

## This study

The National Health and Nutrition Examination Survey (NHANES) is an ongoing series of studies conducted by the Centers for Disease Control (CDC) to assess the health and nutritional status of adults and children in the United States using interviews, physical exams, and laboratory assays. Approximately 5000 participants of all ages are examined each year and data are released in two-year cycles. The study design permits inferences about the civilian, non-institutionalized U.S. population. Some variables are measured every year, and others only in some years. We used the NHANES 2011–2012 data series as it included both grip strength and a depression measure. NHANES oversamples target populations, and the sampling plan for those years strove for specified reliability in sex-age groups for non-Hispanic black persons, non-Hispanic non-black Asian persons, Hispanic persons, and low- and non-low-income groups for the remainder of the U.S. population [56, 57]. Our evolutionary predictions should hold equally for all current populations, however.

## Measures

All measures were from the NHANES 2011–2012 data series. We provide NHANES variable names to permit others to easily replicate our analyses and acquire further information on each variable from the online NHANES documentation [58].

### Response variables: depression and suicidal ideation

The Patient Health Questionnaire (PHQ-9) is a 9-item depression screening instrument that scores each of the 9 DSM-IV criteria for MD from 0 (not at all) to 3 (nearly every day). A depression score is computed by summing items, and a score of ≥10 has good specificity and sensitivity for MD [59]. In the public data series, depression scores were available for ages 18–80 (due to identification risk, depression scores for adolescents are not publicly available, and all NHANES participants older than 80 have age set to 80). Per our predictions (below), we limited our analysis to participants ≤60. We computed suicidal ideation (yes/no) as any non-zero response to DPQ090 (‘Thoughts that you would be better off dead or of hurting yourself in some way’).

### Explanatory variables: grip strength and sex

Grip strength, an index of upper body strength and physical formidability, was measured three times on each hand using a dynamometer. We used combined grip strength, which was the sum of the highest reading of each hand (kg; variable ‘MGDCGSZ’ in the NHANES data). Sex (male/female) was available for each participant.

### Potentially confounding variables

Fortuitously, the NHANES data series included several demographic and health-related variables that might confound the relationships between sex, strength and depression.

Height and weight are positively correlated with grip strength [60], and overweight and obesity are positively correlated with depression [61]. We therefore included standing height (cm; BMXHT) and weight (kg; BMXWT) as potential anthropometric confounds. We computed obese as BMI≥30, where BMI is body mass index ($kg/m^{2}$; BMXBMI). Age was in years (RIDAGEYR).

Socioeconomic position and education are positively associated with adult grip strength and other indices of adult physical capability [62, 63], and are negatively associated with depression [64, 65]. As potential socioeconomic confounds we therefore included adult education level, the highest grade level or degree received (1: less than 9th grade; 2: some high school; 3: high school grad/GED; 4: some college or AA degree; 5: college graduate or above; DMDEDUC2); income, the ratio of family income to regional poverty level, adjusted for family size (0–5; INDFMPIR) and living alone (one person living in the household; DMDHHSIZ = 1), which is a risk factor for depression [66].

Hormones can have strong effects on mood and muscle mass. Testosterone promotes muscle growth, with effects dependent on sex, age, strength-training exercise and administration of supraphysiological doses [67, 68]. Testosterone also appears to have a suppressive effect on depression [45, 69]. Thyroid hormones have a well-known association with depression [70], and hypothyroidism and hyperthyroidism are associated with neuromuscular dysfunction [71]. NHANES 2011–12 included serum total testosterone (ng/dl; LBXTST) and serum thyroid hormones: free thyroxine (T4 free) (ng/dl; LBXT4F) and thyroid-stimulating hormone (TSH) (uIU/ml; LBDTSH1S). Thyroid measures were only available for one third of the sample.

Physical health is associated with both grip strength [72] and depression [73]. NHANES contains hundreds of variables related to physical health. We chose white blood cell count (LBXWBCSI), an indicator of infection; hemoglobin levels (LBXHGB); a physical disability score that reflected difficulty completing 14 physical actions, such as standing up or climbing stairs, which was only available for ages 20–60 (see Supplementary text); and two general self-report measures: number of days in the last month that physical health was not good (0–30; HSQ470) and perceived over or under weight (WHQ030, recoded as normal/abnormal).

### Statistical analysis

NHANES uses a complex, multistage, probability sampling design to select participants representative of the civilian, non-institutionalized US population. Sample weights for each 2-year survey cycle take into account survey non-response, over-sampling, post-stratification and sampling error [56, 57]. We therefore analysed data using R version 3.1.0 (2014-04-10) and the ‘survey’ package, version 3.30-3 [74]. Continuous explanatory and control variables were centered at their means and divided by 2 SD so that regression coefficients represent a 2 SD change, roughly from ‘low’ to ‘high’ values, and are directly comparable to those of binary variables with equal class probabilities, such as sex [75]. For logistic regressions, our tables report coefficients as log odds but in the text we report them as odds ratios (OR), which are easier to interpret. If an analysis contains a variable(s) with missing values, the ‘survey’ package uses domain estimation on the subset of complete cases to recover the population representativeness.

Our main research question was whether the sex difference in grip strength explains the sex difference in depression. In the case that our theoretical predictions (below) are verified, it is of interest to provide a formal numeric estimate for the proportion of sex effects on depression that is mediated by grip strength. We used the ‘mediation’ package for R [76] to derive such estimates (with robust standard errors and 2000 quasi-Bayesian Monte Carlo draws), under the assumption that the effects of strength on depression are causal.

We did not use survey weights in mediation analyses because to our knowledge this is not yet possible for binary outcomes. However, the ‘randomized treatment’ (allocation of sex at birth) is likely to be independent of potential outcomes and mediators (depression and strength, resp.) as required for the validity of analysis [76]. Furthermore, using survey weights is not necessarily advisable when the goal is causal estimation rather than descriptive statistics of a target population [77]. While neglecting survey weights does not affect estimation of the causal effects, it is theoretically possible that our estimate of the average causal mediation effect differs from the population average to some extent.

Our data analysis R code is available at https://bitbucket.org/grasshoppermouse/strengthdepression.

### Predictions

Based on the above considerations we predicted that among physically healthy individuals:

Depression score (PHQ-9), depressed status (PHQ-9 ≥10), and suicidal ideation (DPQ090) will be negatively correlated with grip strength, controlling for age and sex.

After controlling for grip strength, sex will be a weaker predictor of depression (i.e. physical strength will account for much of the sex difference in depression).

Because of the sex-dimorphism in strength (strength having a bi-modal distribution), we expect strategic differences between the clusters, such that there will be an interaction between grip strength and sex, where grip strength will be a stronger negative predictor for men than for women.

Each of the above relationships will persist after controlling for indices of physical health (i.e. the relationship between grip strength and depression will not be entirely due to the relationship between depression and poor physical health).

These predictions are limited to individuals aged ∼18–60 because physical strength increases dramatically during adolescence and declines in old age.

## Results

There were 2115 adult women and 2077 adult men in the study. Using survey weights to estimate population proportions, 8.65% were depressed, 9.72% were living alone, 34.4% were obese (BMI ≥30) and 57.4% perceived their weight to be too high or too low. See Table 1 for summary statistics of continuous variables.

**Table 1.** Summary statistics of continuous variables by sex

Female summary statistics					
Variable	*N*	Min	Max	Mean	SD
Age (years)	2115	18.00	60.00	39.200	12.300
Depression score (PHQ-9)	1710	0.00	27.00	3.510	4.570
Education level	1968	1.00	5.00	3.850	1.120
Ratio of income to poverty level	1950	0.00	5.00	2.780	1.730
Height (cm)	2015	135.00	185.00	163.000	7.210
Weight (kg)	2013	34.70	216.00	76.400	20.700
Body mass index (kg/m-sqr)	2012	13.60	80.60	28.800	7.590
Combinded grip strength (kg)	1779	20.20	104.00	59.200	10.500
Testosterone (ng/dl)	1867	0.74	379.00	25.700	23.200
Free thyroxine (ng/dl)	605	0.43	1.57	0.819	0.128
Thyroid-stimulating hormone (TSH) (uIU/ml)	605	0.22	55.50	2.080	3.180
Days of poor physical health in last month	1655	0.00	30.00	3.080	7.270
White blood cell count (1000 cells/ul)	1946	2.80	15.90	7.190	2.090
Hemoglobin (g/dl)	1946	6.10	19.60	13.300	1.160
Physical disability score	1967	0.00	14.00	0.857	2.480

Male summary statistics					
Variable	N	Min	Max	Mean	SD
Age (years)	2077	18.000	60.00	38.900	12.700
Depression score (PHQ-9)	1782	0.000	26.00	2.760	4.130
Education level	1920	1.000	5.00	3.690	1.160
Ratio of income to poverty level	1899	0.000	5.00	2.830	1.710
Height (cm)	1983	140.000	204.00	176.000	7.600
Weight (kg)	1982	39.600	204.00	88.400	20.300
Body mass index (kg/m-sqr)	1981	15.800	66.20	28.400	6.060
Combinded grip strength (kg)	1828	21.900	170.00	93.000	17.100
Testosterone (ng/dl)	1828	2.700	1400.00	411.000	171.000
Free thyroxine (ng/dl)	617	0.500	1.78	0.828	0.148
Thyroid-stimulating hormone (TSH) (uIU/mL)	617	0.035	18.50	1.820	1.510
Days of poor physical health in last month	1750	0.000	30.00	2.640	6.690
White blood cell count (1000 cells/ul)	1892	1.700	16.80	6.940	2.030
Hemoglobin (g/dl)	1892	8.500	19.00	15.100	1.090
Physical disability score	1920	0.000	14.00	0.678	2.150

Top: females. Bottom: males. Mean and SD were computed using survey weights, and therefore represent population estimates

### Strength and depression

In Table 2, models 1–4 are survey-weighted logistic regression models of depressed status; models 5–8 are survey-weighted linear regression models of depression scores and model 9 is a survey-weighted logistic regression model of suicidal ideation (present/absent).

**Table 2.** Models of depression as functions of sex, age, grip strength, and interactions

Dependent variable									
	Depressed status				Depression score				Suicidal *Survey-weighted logistic*
	*Survey-weighted logistic*				*Survey-weighted normal*				
Term	Model 1	Model 2	Model 3	Model 4	Model 5	Model 6	Model 7	Model 8	Model 9
Age (s)	0.308*	0.185	0.184	0.0402	0.268	0.0446	0.0453	0.0244	−0.0595
	(0.0236, 0.593)	(−0.0901, 0.46)	(−0.093, 0.461)	(−0.256, 0.336)	(−0.094, 0.63)	(−0.341, 0.43)	(−0.343, 0.434)	(−0.349, 0.398)	(−0.569, 0.45)
Female	0.505**	−0.164	−0.184	−0.171	0.745**	−0.0308	−0.0147	−0.0436	0.0244
	(0.252, 0.758)	(−0.681, 0.352)	(−0.765, 0.397)	(−0.692, 0.349)	(0.35, 1.14)	(−0.678, 0.616)	(−0.679, 0.65)	(−0.689, 0.602)	(−0.601, 0.649)
Strength (s)		−0.91**	−0.874*	−0.909**		−1.04**	−1.06*	−1.06**	−0.629
		(−1.49, −0.334)	(−1.57, −0.176)	(−1.49, −0.323)		(−1.69, −0.387)	(−1.93, −0.192)	(−1.71, −0.415)	(−1.29, 0.0355)
Female:Strength			−0.105				0.0875		1.22**
			(−1.29, 1.08)				(−1.62, 1.79)		(0.436, 2.01)
Age:Strength				−0.843**				−0.505	
				(−1.33, −0.356)				(−1.17, 0.164)	
Intercept	−2.64***	−2.39***	−2.4***	−2.42***	2.76***	3.06***	3.07***	3.06***	−3.2***
	(−2.93, −2.35)	(−2.77, −2.01)	(−2.76, −2.04)	(−2.8, −2.03)	(2.38, 3.14)	(2.55, 3.57)	(2.56, 3.59)	(2.55, 3.57)	(−3.68, −2.72)
Observations	3492	3258	3258	3258	3492	3258	3258	3258	3262
AIC	2169	1958	1962	1950	70 420	61 970	62 080	61 970	1002

Models 1–4: logistic regression models of depressed status as functions of our primary explanatory variables. Models 5–8: linear regression models of depression score. Model 9: logistic regression model of suicidal ideation (present/absent). Variables with (s) were centered at their means and standardized by 2 SD. Logistic regression coefficients are log odds (95% CI). All models were fitted using survey weights. AIC values can only be compared among models with the same outcome variable and sample size.

***P < 0.001; **P < 0.01; *P < 0.05

Model 1 (Table 2) simply tested whether there was a sex difference in depressed status. As seen in most studies of depression, female sex was positively associated with depressed status (OR = 1.66, *P* = 0.0014).

Model 2 (Table 2) added grip strength to model 1 to test predictions 1 and 2. Strength attenuated the effect of sex on depressed status from an odds ratio of 1.66 to odds ratio of 0.848, corresponding to a 133 percentage unit change in the regression coefficient (i.e. change of sign). The estimated association between strength and depressed status, adjusting for sex, was nearly twice as strong in absolute magnitude as the original association between sex itself and depressed status (OR = 0.4, *P* = 0.008).

Model 3 (Table 2) added an interaction between sex and strength to model 2 to test prediction 3. Contrary to predictions, there was no significant interaction of sex and strength on depressed status (*P* = 0.86, Table 2, model 3), and the main effect of strength on depressed status remained virtually unchanged from model 2.

**Figure 2.**
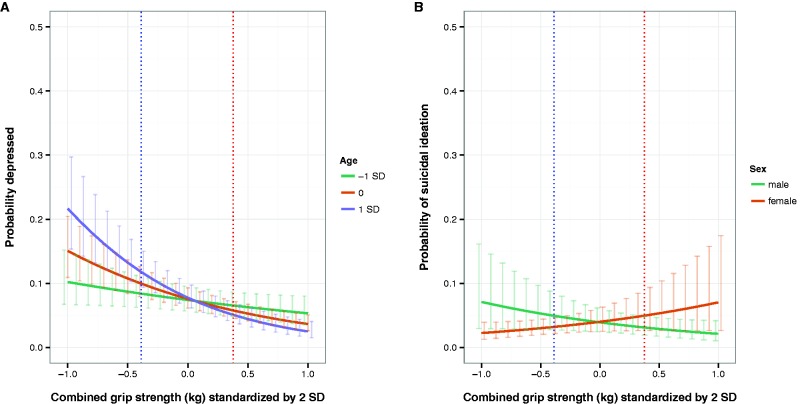
A: The effect of grip strength on depressed status for −1 SD age (27 years), mean age (39 years), and +1 SD age (52 years) across both males and females. B: The relationship between suicidal status and combined grip strength with interaction by sex. Plotted at age = 39. Dotted vertical lines are mean female strength (blue) and mean male strength (red). See Table 2, models 4 and 9. The range of the *x*-axes is ± 2 SD

Model 4 (Table 2) added an interaction between age and strength to model 2. There was unexpected significant interaction, such that the effect of strength on depressed status increased with age (see Fig. 2A).

Models 5–8 (Table 2) repeated the foregoing analyses, except using survey-weighted linear regression with depression score as the outcome variable, with qualitatively similar results. Sex was a significant predictor of depression score (model 5), but was no longer significant after entering grip strength, which was a significant predictor (model 6), supporting predictions 1 and 2. Model 7 failed to find a significant interaction between sex and strength, contrary to prediction to prediction 3. Model 8 found that age interacted with strength, such that the effect of strength on depression score increased with age, similar to model 4, but this effect was not significant.

In a non-weighted analysis, strength, age and sex explained 1.5% of the variance in PHQ-9 score, but adding grip strength to the model changed the sex coefficient only by 40.7 percentage units from the original. In a survey-weighted analysis of the PHQ-9 score, the change was 104 percentage units. We will later discuss this discrepancy in light of the results below and the fact that NHANES weights older participants between 40 and 60 years more heavily (undersamples relative to population) compared with younger participants (18–40 years) in our target sample, having a weight by age correlation of 0.085 (*P* = 3.9 × 10 ^−^^8^).

Model 9 (Table 2) tested predictions 1–3 for suicidal ideation. Contrary to our prediction, there was no significant main effect for strength (*P* = 0.34) as a predictor of suicidal ideation, controlling for sex and age. However, partially consistent with prediction 3, there was a significant interaction of sex and strength, with strength a negative predictor of suicidal ideation for men, as predicted, but a positive predictor for women, contrary to predictions (see Fig. 2B).

As men are much stronger than women (Fig. 1), it is theoretically important to ascertain that strength is not simply a proxy for gender when modeling depression. Accordingly, we did not observe a significant interaction between sex and strength on depressed status (model 3, Table 2), and strength had similar effects on depressed status both in men (OR = 0.5, *P* = 0.021) and in women (OR = 0.61, *P* = 0.048).

### Controlling for potential confounds

**Figure 3.**
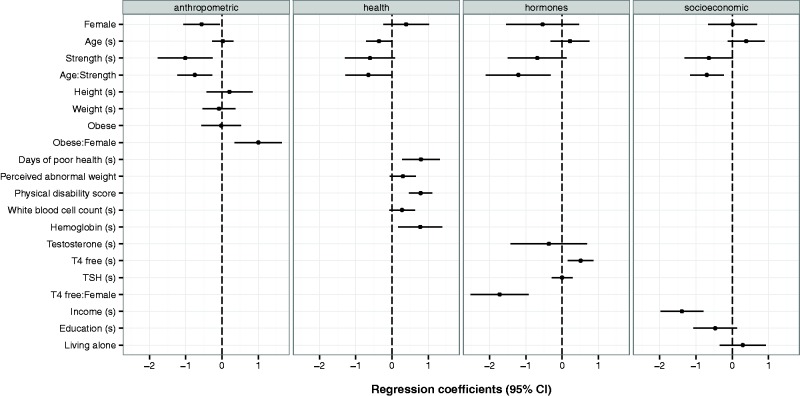
Coefficient plot for logistic regression models of depressed status among adults aged 18–60 for different groups of control covariates. Variables with (s) have been centered at their means and standardized by 2 SD. Coefficients are log odds with 95% CI. See Supplementary Table S1 for numeric values. All models fit using survey weights

Models with four groups of potential confounds are displayed in Fig. 3 (see Supplementary Table S1 for numeric estimates). Partially in line with our prediction 4, the effects of grip strength on depression, or its interaction with age, persisted when controlling for three of the four groups: anthropometric, hormones and socioeconomic. After controlling for variables in the health group, however, the effects of strength were in the predicted direction, but no longer significant. We explore this group of variables in more detail below.

In addition, female obesity, free thyroid hormones and income level were important independent predictors of depression. Among the non-health covariate groups, adjusting for socioeconomic factors attenuated the effect of strength the most, dropping the direct effect of strength to 70.6% of the original association in Table 1, and its age-interaction effect to 82.8% compared to the original.

### Exploratory analysis of health covariates

Partially contrary to prediction 4, the effects of strength on depressed status were not significant after controlling for the health-related covariates (Fig. 3; but see the statistical note in the supplementary document). We therefore conducted exploratory analyses to determine (i) if including the strength terms nevertheless improved the fit of the health model; (ii) if the health covariates also accounted the effect of sex on depressed status, completely contrary to prediction 4 and (iii) which health covariate(s) most confounded the strength effects.

According to the Akaike information criterion (AIC) [78], including strength and strength-by-age terms in the health model improved fit relative to a model without these terms (AIC = 1414.9 vs AIC = 1418.6). In a health model excluding only the strength terms, female sex was a strong positive predictor of depressed status (OR = 2.32, *P* = 0.0063), indicating that the health covariates did not account for the effect of sex on depressed status (model 5, Supplementary Table S1); in contrast, sex was not a significant predictor of depressed status in any model with the strength terms.

The health-related variables with the strongest bivariate correlations with strength were hemoglobin (*r* = 0.52) and disability score (*r* = −0.14). (For the full correlation matrix, see Supplementary Table S2.) The strong correlation of hemoglobin and strength was partially due to a sex difference in hemoglobin levels; additionally, there was an interaction, such that hemoglobin was a positive predictor of male, but not female, strength (model not reported). Starting with a model of depressed status as a function of sex, age, strength, and the age-strength interaction, we alternatively entered hemoglobin or disability score. Disability score reduced the strength coefficients the most: the main effect of strength was 62.6% the original, and the age-strength interaction term was 70.1% of the original (model 6, Supplementary Table S1).

Finally, disability appeared to interact with strength: the effect of strength on depressed status was greatest among those with little or no disability (model 7, Supplementary Table S1). This is consistent with the bargaining model because disability can reduce fighting ability independent of upper body strength (e.g. wheelchair bound individuals might have high grip strength but low fighting ability).

### Proportion of the sex difference mediated by strength

We found little evidence that the effect of grip strength on depression was best explained as a confound with factors related to age, anthropometrics, hormones or socioeconomics, but was partially explained by a confound with physical disability. As noted above, that shared variance may nevertheless reflect shared differences in ability to pose a physical threat to others. Given the arguments presented in the Introduction, we proceeded to estimate the proportion of the sex effect on depression that is mediated by grip strength under causal mediation [76]. That is, model 4 of Table 2 is the outcome model, and an ordinary linear model predicting grip strength with age and sex the mediation model. This is an instance of ‘moderated mediation’, where the amount of mediation can differ in different age groups.

**Figure 4.**
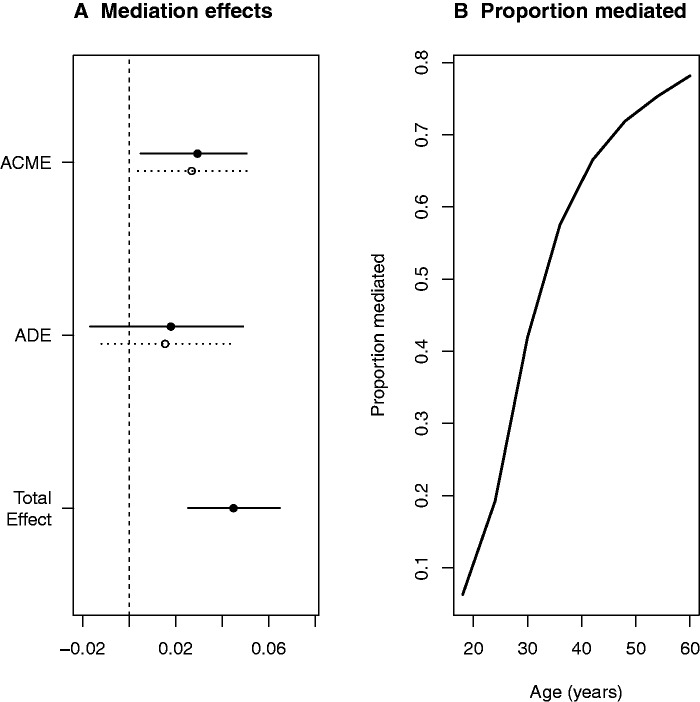
Mediation of sex effect on depression by strength. (A) Average mediation effects. Estimated mediation effects were similar for both men (open circles; dotted line for 95% CI) and women (closed circles; solid line for 95% CI). (B) Moderated mediation. Estimated proportion of the total sex effect that is mediated by strength, given as a function of age (the moderating variable). ACME, average causal mediation effect; ADE, average direct effect. Notice that Total Effect is ACME + ADE, averaged over sexes, or ‘treatments’. Proportion mediated is average ACME divided by the total effect; interpretation of the proportion is straightforward only when ACME and ADE are of the same sign

Overall, in this non-weighted analysis, we estimated that female sex increased depression prevalence by 4.5 percentage units compared with men (6.8% prevalence), and altogether 63% of that total effect of sex on depression was mediated by grip strength (CI = 10–150% and Pensp;=ensp;0.021 for mediated effect; the upper confidence interval exceeding 100% represents the possibility that the direct effect is negative). The observed mediation effect was practically the same when adjusting for the quadratic and cubic effects of age on strength (63% mediated, Pensp;=ensp;0.027). Figure 4 shows this decomposition of the total effect, but also how strongly the mediation effect depended on age of the participant (Pensp;=ensp;0.002 for age interaction in the mediated effect between the 18 and 60 years old subjects).

For comparison, when analyzing free T4 in the exact same way as we did for strength, we found only a direct effect [average direct effect (ADE) = 0.039, Pensp;=ensp;0.026] and no mediation effect [average causal mediation effect (ACME) = 0.0015, Pensp;=ensp;0.21]. When analyzing testosterone in the same way as we did for strength, we found neither a statistically significant direct effect (ADE = 0.036, Pensp;=ensp;0.085) nor a mediated effect (ACME = 0.0087, Pensp;=ensp;0.66), despite finding a highly significant total effect (0.045, Pensp;<ensp;0.001). Income did not show a significant mediating effect (Pensp;=ensp;0.2), and hemoglobin had opposite mediated (ACME = −0.026, Pensp;=ensp;0.003) and direct (ADE = 0.067, Pensp;<ensp;0.001) effects, meaning that being a female reduced its effect on depression in comparison to males.

The survey-weighted female bias in disability was very small (*d* = 0.077). In an unweighted analysis, disability score mediated 11% of the sex effect on depression (CI = 1.8–25%, Pensp;=ensp;0.016), which was small compared to the estimate for strength.

## Discussion

The unified bargaining model of anger and depression proposes that when the behavior of social partners is costly to the individual, such as when adversity strikes and sufficient help is not forthcoming, the physically strong are liable to become overtly angry, whereas the physically weak are liable to become depressed and suicidal. Our results supported the inverse relationship between upper body strength and depressed status and depression score, as seen in previous studies, and additionally indicated that the well-established sex difference in depression is due, in large part, to the marked sexual dimorphism in upper body strength. A 2 SD increase in grip strength reduced the odds of depressed status by more than half (OR = 0.4; Table 2, model 2). Controlling for grip strength, sex was not a significant predictor of depressed status, and males were slightly more likely to be depressed than females. We estimated that on average 63% of the sex effect in depression was due to the sex differences in strength. Contrary to predictions, there was no significant interaction between sex and strength. Grip strength was also a significant predictor of suicidality, with an interaction with sex such that grip strength was a negative predictor of suicidality for males, as predicted, but a positive predictor for females, contrary to predictions.

We unexpectedly found that, for both sexes, the effect of strength on depressed status was stronger at older ages. In older adults, up to 78% of the sex effect may due to strength. In an evolutionary game theory analysis in which younger individuals do not yet know their own strength relative to others whereas older individuals have learned their relative strength from a history of wins and losses, strength had a weaker effect on the choice to fight in the young and a stronger effect on the choice in the old [79]. Under the unified bargaining model, perhaps strong older individuals ‘choose’ overt physical threats to resolve severe disputes, whereas weaker older individuals ‘choose’ depression (with no conscious choice implied).

Our finding that sexual dimorphism in strength appears to explain the sex difference depression could help explain the emergence of sex differences in MD during adolescent because the sexual dimorphism in strength also emerges during adolescence (Fig. 1). The age moderation effect might speak against this hypothesis, however. NHANES depression scores are publicly available only for individuals ≥ 18 years old, so we could not test this hypothesis in this study.

We also found that the effects of grip strength on depressed status were partially confounded with health-related variables, especially physical disability, which exhibited a small but significant female bias. Hence, low strength could be a risk factor for depression because it is a proxy for poor health rather than low fighting ability, contrary to the bargaining model. Exploratory analyses of the health variables found that without strength in the model, sex was a strong and highly significant predictor of depressed status (model 5, Supplementary Table S1), whereas including the strength terms improved overall model fit and the effect of sex became small and not significant (models 4 and 6, Supplementary Table S1). An alternative interpretation is therefore that physical disability is also, in part, a largely sexually monomorphic index of low fighting ability, which is supported by the apparent interaction of strength and disability as risk factors for depression, with a greater effect of strength at lower levels of disability (model 7, Supplementary Table S1).

Contrary to estimation of target-population parameters, it is not always clear whether estimation and interpretation of causal effects in complex survey data is better done with or without the use of sample weights, and therefore trying both has been suggested as a sensitivity analysis [77]. Indeed, we found that strength attenuated the sex difference more fully in the weighted sample than in the non-weighted sample, meaning that our proportion-mediated estimate may be conservative. The above-discussed age-interaction is likely to explain this finding: because NHANES weights older people more heavily than young people in our sub-sample (participants between 18 and 60 years), and strength accounts for a larger share of the sex difference in depression for older than for younger participants (Fig. 4), the average role of strength appears greater in the weighted sample than in the non-weighted sample.

It is important to emphasize that although strength accounted for much of the effect of sex on depressed status, and that low upper body strength is a risk factor for depression, depression is caused by environmental factors—adverse life events are prime culprits—interacting with genetic factors [80]. Hence, the unified bargaining model does not directly predict ‘kindling’ or depressive-episode cycle acceleration, as in non-adaptive, disorder-based theories of depression [81].

## Limitations

Our cross-sectional study cannot determine causation. Grip strength is only an approximate indicator for success in physical conflict, and NHANES data did not include all factors that are central to the unified bargaining model. The lack of a measure of adversity reflects the lamentable trend to decontexualize depression [82], and there was no measure of conflict or anger. And it is possible, of course, that the mediating role of grip strength is due to a sexually dimorphic confound that we did not examine here, such as some sexually dimorphic aspect of health or well-being, or some sex difference in exposure to depression risk factors, that is unrelated to fighting ability. This study also suffers from the general limitation that the PHQ-9 confounds suicidal ideation with passive thoughts of death or self-harm.

The causal mediation analysis we used relies on the sequential ignorability assumption [76]. The first part of that assumption is quite likely to hold, since the genetic allocation of sex at birth (‘treatment’) has been ‘randomized’ relative to subsequent assessments of strength and depression, controlling for the age of assessment. It is less clear whether all the causal paths (correlations) between depression and strength have been taken into account by the mediation model. Although sensitivity analyses are available for studying the effects of residual correlations on the mediation inferences [76], they are not yet available for the moderated mediation model we used. Techniques for survey weighting in this context are also still lacking. Future studies may alleviate these technical shortcomings; however, full certainty on sequential ignorability is never attainable [76]. Our extensive analysis of NHANES data found little evidence that the mediating role of strength on the sex bias in depression was best explained as a confound (we did not explicitly study all the variables in the mediation model, since many of them were only recorded for much smaller sub-samples and were not easily interpretable as ‘pre-treatment’ confounders).

## Concluding remarks

This empirical study underscores two important and general theoretical points for the study of behavioral traits. First, theory shows that understanding the emergence of consistent behavioral traits, e.g. disposition for depression, may require considering their joint evolution and development with physiological traits, e.g. physical strength [83].

Second, our study highlights the importance of constructing explicit game-theoretical models for the interactions among individual behavioral traits [17, 83] in which different behavioral traits can be seen as competing strategies that solve the same problem; thus, a behavioral trait or strategy does not exist in a social ‘vacuum’ but is dynamically determined by the other strategies that are present. For example, risk of depression could depend on the levels of aggression, deceit and cooperation in one’s local social environment [17]. In such cases, isolated studies of individual traits can achieve only limited progress. Our theory on the interplay of aggressive and depressive tendencies yielded an explanation for the seemingly unrelated and puzzling findings on sex differences in depression.

In summary, we found that grip strength, an index of upper body strength and physical formidability, is a risk factor for depression, albeit one that overlaps with physical disability. Grip strength mediates much of the effect of sex on depression, however, whereas disability mediates a small fraction. Our results cannot rule out other evolutionary theories of MD, or the mainstream view of MD as a brain dysfunction. Nonetheless, in light of the relationships between strength, anger and aggression, and between anger, conflict and depression, our results lend support to the idea that depression, suicidality, and other forms of self-harm [36] are strategies that function, in part, to compel reluctant social partners to provide more help or otherwise alter their behavior in ways that benefit powerless victims of adversity.

## Supplementary Material

Supplementary Data
