# Supplementary material for: Explaining the sex difference in depression with a unified bargaining model of anger and depression
Source: Evol Med Public Health. 2016 Feb 15;2016(1):117–32. doi: 10.1093/emph/eow006 (PMC4804352; doi:10.1093/emph/eow006)
Supplement: Supplementary Data [file supp_2016_1_117__index.html]

Explaining the sex difference in depression with a unified bargaining model of anger and depression — Supplementary Data 

# Explaining the sex difference in depression with a unified bargaining model of anger and depression

## Supplementary Data

files

- Supplementary Data - pdf file
