# Supplementary material for: Explaining the sex difference in depression with a unified bargaining model of anger and depression
Source: Evol Med Public Health. 2016 Feb 15;2016(1):117–32. doi: 10.1093/emph/eow006 (PMC4804352; doi:10.1093/emph/eow006)
Supplement: Supplementary Data [file supp_eow006_Supplementary_information.pdf]

Supplementary information

Physical disability items and score

The physical disability score was the number of the following 14 activities that the participant indicated at least some difficulty performing, and thus ranged from 0-14 (only available for ages 20-60):

```
PFQ054  - Need special equipment to walk (weighted double because equiv. to
yes on both 61B and 61C)

or

PFQ061B - Walking for a quarter mile difficulty
PFQ061C - Walking up ten steps difficulty

plus

PFQ061D - Stooping, crouching, kneeling difficulty
PFQ061E - Lifting or carrying difficulty
PFQ061H - Walking between rooms on same floor
PFQ061I - Standing up from armless chair difficulty
PFQ061J - Getting in and out of bed difficulty
PFQ061K - Using fork, knife, drinking from cup
PFQ061L - Dressing yourself difficulty
PFQ061M - Standing for long periods difficulty
PFQ061N - Sitting for long periods difficulty
PFQ061O - Reaching up over head difficulty
PFQ061P - Grasp/holding small objects difficulty
PFQ061T - Push or pull large objects difficulty
```

The following activities were omitted because they could involve cognitive or emotional disability and/or low motivation (which might be confounded with depression):

```
PFQ061A - Managing money difficulty
PFQ061F - House chore difficulty
PFQ061G - Preparing meals difficulty
PFQ061Q - Going out to movies, events difficulty
PFQ061R - Attending social event difficulty
PFQ061S - Leisure activity at home difficulty
```

Model coefficients

term	(1) anthropometric	(2) hormones	(3) socioeconomic	(4) health	(5) health w/o strength	(6) disability	(7) disability2
Female	-0.563 (-1.06, -0.063)	-0.538 (-1.54, 0.46)	0.0135 (-0.658, 0.685)	0.388 (-0.24, 1.02)	0.843** (0.363, 1.32)	0.0171 (-0.498, 0.532)	-0.0201 (-0.537, 0.497)
Age (s)	0.0222 (-0.267, 0.312)	0.217 (-0.315, 0.748)	0.386 (-0.125, 0.897)	-0.36 (-0.712, -0.00863)	-0.193 (-0.457, 0.071)	-0.269 (-0.567, 0.0281)	-0.341 (-0.657, -0.026)
Strength (s)	-1.01* (-1.76, -0.259)	-0.684 (-1.49, 0.126)	-0.642 (-1.31, 0.0235)	-0.609 (-1.3, 0.0804)		-0.569 (-1.2, 0.0633)	-0.712 (-1.41, -0.0112)
Age:Strength	-0.747* (-1.23, -0.27)	-1.21* (-2.1, -0.315)	-0.698* (-1.16, -0.23)	-0.653 (-1.29, -0.016)		-0.591* (-1.08, -0.096)	-0.859* (-1.55, -0.164)
Height (s)	0.207 (-0.426, 0.839)						
Weight (s)	-0.0824 (-0.531, 0.366)						
Obese	-0.0217 (-0.565, 0.522)						
Obese:Female	1.0* (0.349, 1.65)						
Days of poor health (s)				0.795* (0.274, 1.31)	0.788* (0.279, 1.3)		
Perceived abnormal weight				0.297 (-0.0627, 0.657)	0.281 (-0.0763, 0.638)		
Disability score (s)				0.786** (0.467, 1.1)	0.839*** (0.549, 1.13)	1.14*** (0.912, 1.37)	1.28*** (1.04, 1.53)
Strength:Disability							0.542 (0.000673, 1.08)
White blood cell count				0.276 (-0.0776, 0.629)	0.25 (-0.102, 0.601)		
Hemoglobin (s)				0.777* (0.169, 1.39)	0.76* (0.147, 1.37)		
Testosterone (s)		-0.366 (-1.41, 0.682)					
T4 free (s)		0.509* (0.152, 0.866)					
TSH (s)		-0.00103 (-0.29, 0.287)					
T4 free:Female		-1.72** (-2.52, -0.92)					
Income (s)			-1.39*** (-1.97, -0.797)				
Education (s)			-0.469 (-1.07, 0.13)				

Living alone			0.29<br>(-0.344, 0.924)				
Intercept	-2.44***<br>(-2.89, -1.99)	-2.37***<br>(-3.11, -1.64)	-2.72***<br>(-3.23, -2.22)	-3.12***<br>(-3.61, -2.63)	-3.31***<br>(-3.75, -2.86)	-2.6***<br>(-2.99, -2.22)	-2.6***<br>(-2.98, -2.22)
Observations	3235	988	2791	2794	2794	2983	2983
AIC	1916	589.9	1669	1415	1419	1717	1713

Table S1: Logistic regression models of depressed status as functions of sex, age, and grip strength, controlling for groups of potentially confounding variables. Coefficients are log odds (95% CI). All models fit using survey weights. Variables with (s) were standardized by 2 SD. AIC values can only be compared among models with the same sample size. ***p<.001; **p<.01; *p<.05.

Correlation matrix of strength and health variables

	strength	days_poor_health	disability_score	whitebloodcell	hemoglobin	age
strength	1.000	-0.083	-0.140	-0.068	0.520	-0.100
days_poor_health	-0.083	1.000	0.410	0.069	-0.037	0.130
disability_score	-0.140	0.410	1.000	0.110	-0.026	0.190
whitebloodcell	-0.068	0.069	0.110	1.000	0.072	-0.025
hemoglobin	0.520	-0.037	-0.026	0.072	1.000	0.017
age	-0.100	0.130	0.190	-0.025	0.017	1.000

Table S2: Correlation matrix of strength and health-related covariates.

Statistical note

In the health model, the p-value for the age*strength term reported by the survey package is 0.08, but the 95% CI reported by the survey package for this term excludes 0. We extensively investigated this inconsistency, which turns on the default denominator degrees of freedom. The survey glm routine defaults to a value that is "recommended by Korn and Graubard and is correct for PSU-level covariates but is potentially very conservative for individual-level covariates. To get tests based on a Normal distribution use df.resid=Inf." The survey confidence interval routine, in contrast, defaults to df.resid=Inf. NHANES has about 15 county-based primary sampling units (PSUs) per year, for a total of 30 units in the two-year series. However, age and strength are individual-level covariates, which would suggest that the normal approximation might be more suitable; the normal approximation yields a p-value of 0.04 for the age*strength term. Nevertheless, we report the more conservative non-significant default value (p=0.08) in the body of the paper (but report the default 95% CI in the supplementary material).
